# Supplementary material for: Cnidarian-bilaterian comparison reveals the ancestral regulatory logic of the β-catenin dependent axial patterning
Source: Nat Commun. 2021 Jun 29;12:4032. doi: 10.1038/s41467-021-24346-8 (PMC8241978; doi:10.1038/s41467-021-24346-8)
Supplement: Supplementary file 1 — Supplementary Information [file 41467_2021_24346_MOESM1_ESM.pdf]

Supplementary Information

Cnidarian-bilaterian comparison reveals the ancestral regulatory logic of the β-catenin dependent axial patterning

Tatiana Lebedeva, Andrew J. Aman, Thomas Graf, Isabell Niedermoser, Bob Zimmermann, Yulia Kraus, Magdalena Schatka, Adrien Demilly, Ulrich Technau and Grigory Genikhovich

Contents

Supplementary Figures 1-10

Supplementary Results and Discussion 1-3

Supplementary Tables 1-5

Supplementary References

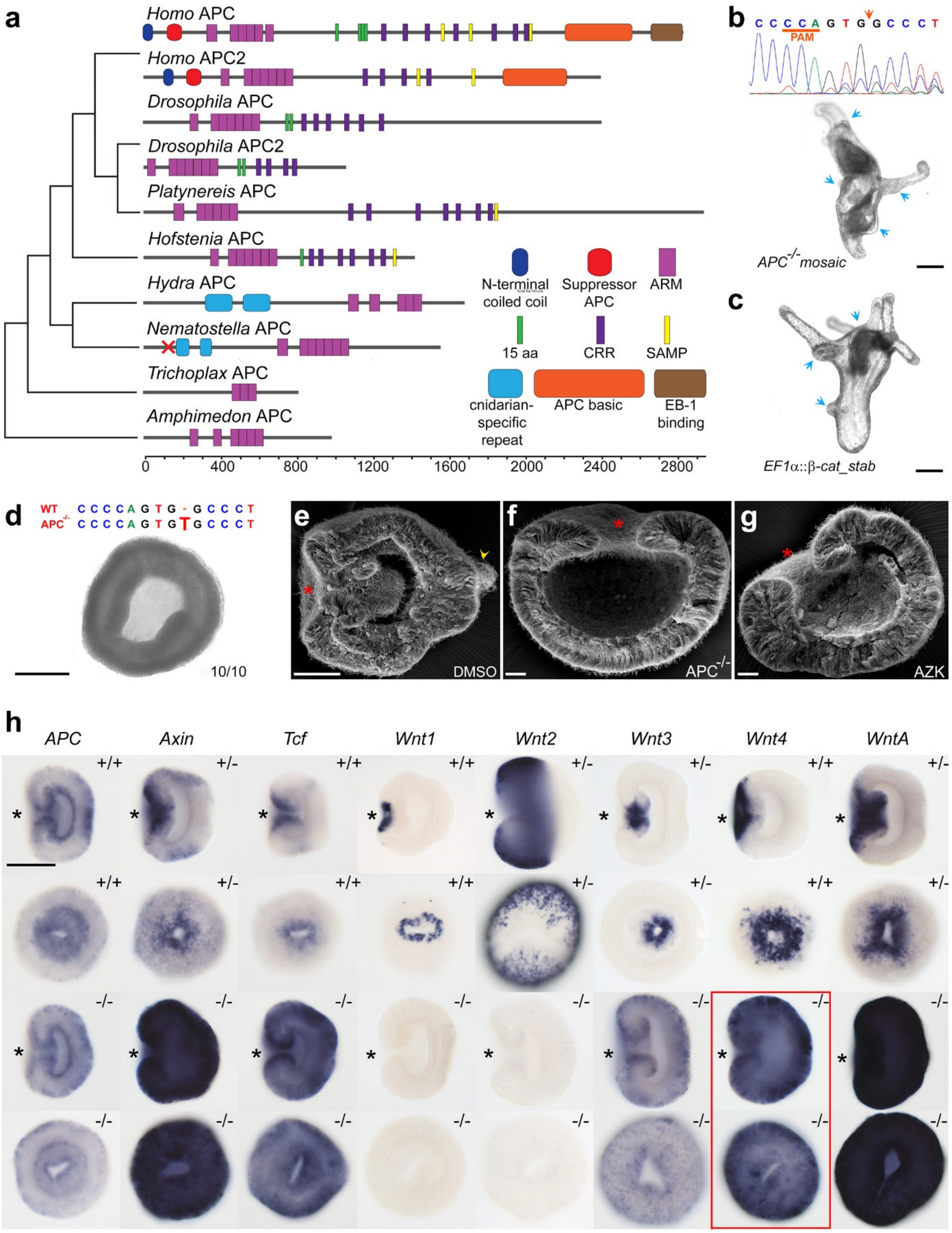

Supplementary Fig. 1: Characterization of the *APC* mutants. a, SMART annotation[1] of the domain structure of the animal APC proteins. Cnidarian APCs have armadillo repeats (ARM), but appear to be missing the typical 15 and 20 amino acid repeats (15 aa, CRR) present in Bilateria and used for β-catenin binding. SAMP – Axin binding domain. Red cross on the *Nematostella* protein indicates the position of the frameshift mutation. **b, c,** Although

several important domains are missing in the non-bilaterian APC proteins, *Nematostella* APC appears to act via β-catenin. **(b)** Mosaic *APC* mutant develops multiple ectopic oral structures such as mouths and/or tentacles (blue arrows). Upon genotyping of this polyp, the sequencing chromatogram shows the accumulation of extra peaks around the Cas9 cutting site (orange arrow). **(c)** Mosaic expression of the stabilized form of the *Nematostella* β-catenin results in a comparable phenotype. Blue arrows – ectopic oral structures. **d,** Genotyping shows that 10/10 3 dpf F2 embryos demonstrating the oralization phenotype are homozygous *APC* mutants with a T insertion. The image shows a representative *APC* mutant (oral view). **e-g,** SEM image of a control 3 dpf planula **(e)** with an elongated oral-aboral axis, a closed mouth (asterisk), a pharynx, and an apical tuft (arrowhead) compared to a homozygous *APC* mutant **(f)** and an AZK treated embryo **(g)**. The latter two **(f-g)**, show a flattened morphology, no pharynx and a secondarily widely open mouth (asterisk). The representative phenotypes shown on **(e-g)** are observed in all scanned embryos (n>10). **h,** In situ hybridization analysis of *APC* and known "saturating" and "window" genes at the late gastrula stage. Oral views are shown below the corresponding lateral views. The genotype of the embryo is shown in the upper right corner of each photo. +/+ wild type; +/- heterozygous *APC* mutant; -/- homozygous *APC* mutant. *APC* is expressed in the endoderm, the forming pharynx and in a shallow aboral-to-oral gradient in the ectoderm. *Axin* and *Tcf*, in contrast, are expressed in an oral-to-aboral gradient with a second area of stronger expression at the aboral boundary of the midbody domain. *APC* behaves as a saturating gene in the APC mutant (just as *Axin*, *Tcf*, *Wnt3* and *WntA* – as previously described for AZK treatments[2]). *Wnt1* and *Wnt2* behave as window genes in the *APC* mutants and upon AZK treatment[2]. The only discrepancy in the expression behavior was observed in the case of *Wnt4*, which, for a yet unknown reason, behaves as a saturating gene in the *APC* mutant, but as a "window" gene in the AZK[2] (red frame). Lateral views (oral to the left) and oral views are shown. Asterisks on lateral views indicate the blastopore.

Scale bars: b-d, h – 100 μm, e – 50 μm, f-g – 20 μm.

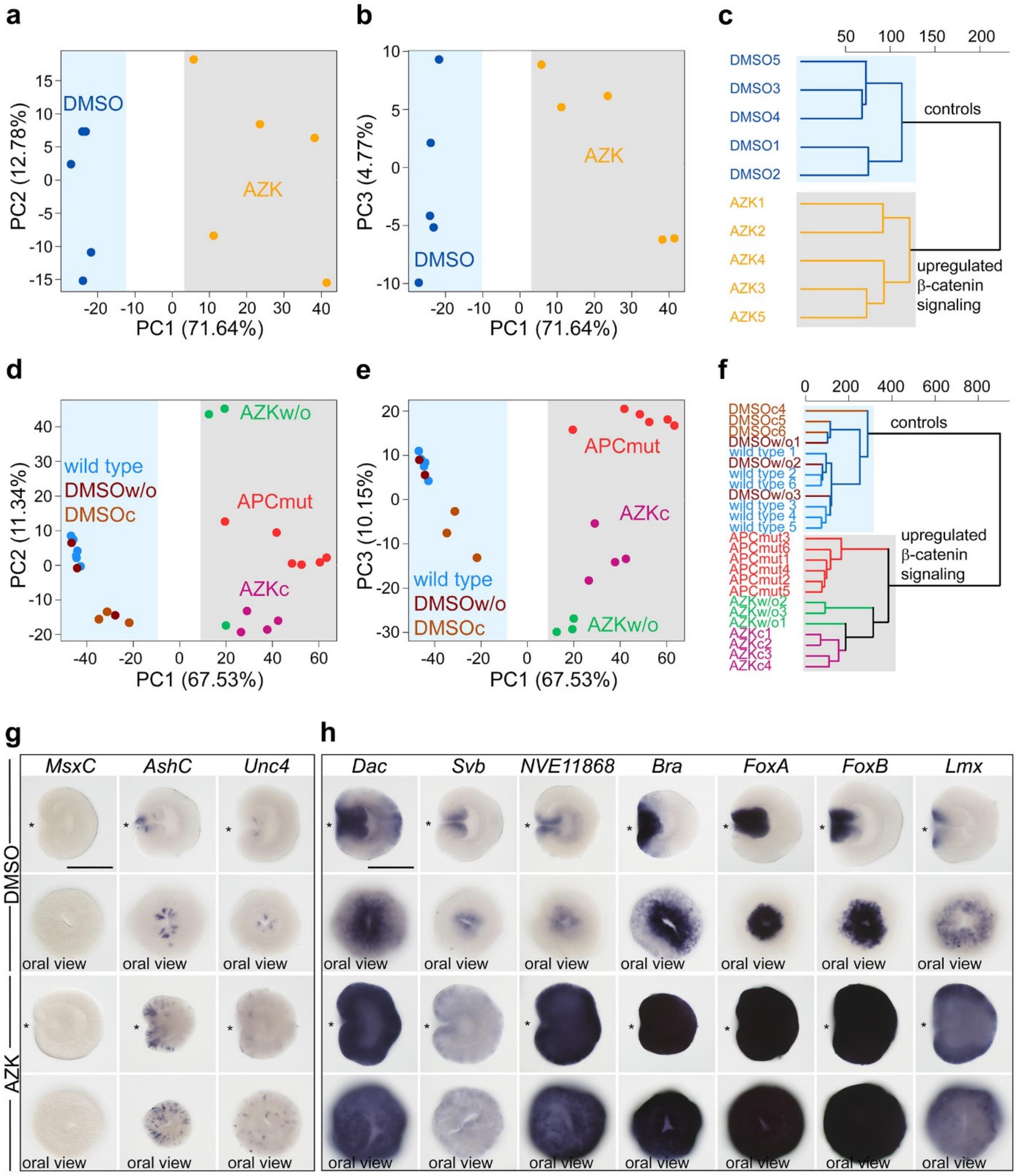

Supplementary Fig. 2: Comparison of transcriptomes after different treatments and expression of the repressor X candidates in DMSO and AZK. a-c, Principle component analysis and a cluster dendrogram clearly separate the transcriptomes of the biological replicates of the AZK treated (grey background) and DMSO treated (blue background) 30 hpf embryos. **d-f,** Principle component analysis and a cluster dendrogram clearly separate the transcriptomes of the biological replicates of the AZK treated embryos and APC mutants (grey background) versus DMSO treated and untreated wild type (blue background) 3dpf

embryos. AZKc – continuous AZK treatment for 3d, AZKw/o – AZK treatment from 10 hpf until 30 hpf followed by a washout for 2 days. **g-h,** Identification of the saturating genes among the ten repressor X candidates. *MsxC* is not detectable at 1 dpf, *AshC* and *Unc4* increase their expression in AZK but are expressed in individual cells **(g)**. The seven remaining genes display a typical saturating phenotype **(h)**. Lateral views (oral to the left) and oral views are shown. Asterisks on lateral views indicate the blastopore. Scale bars 100 μm.

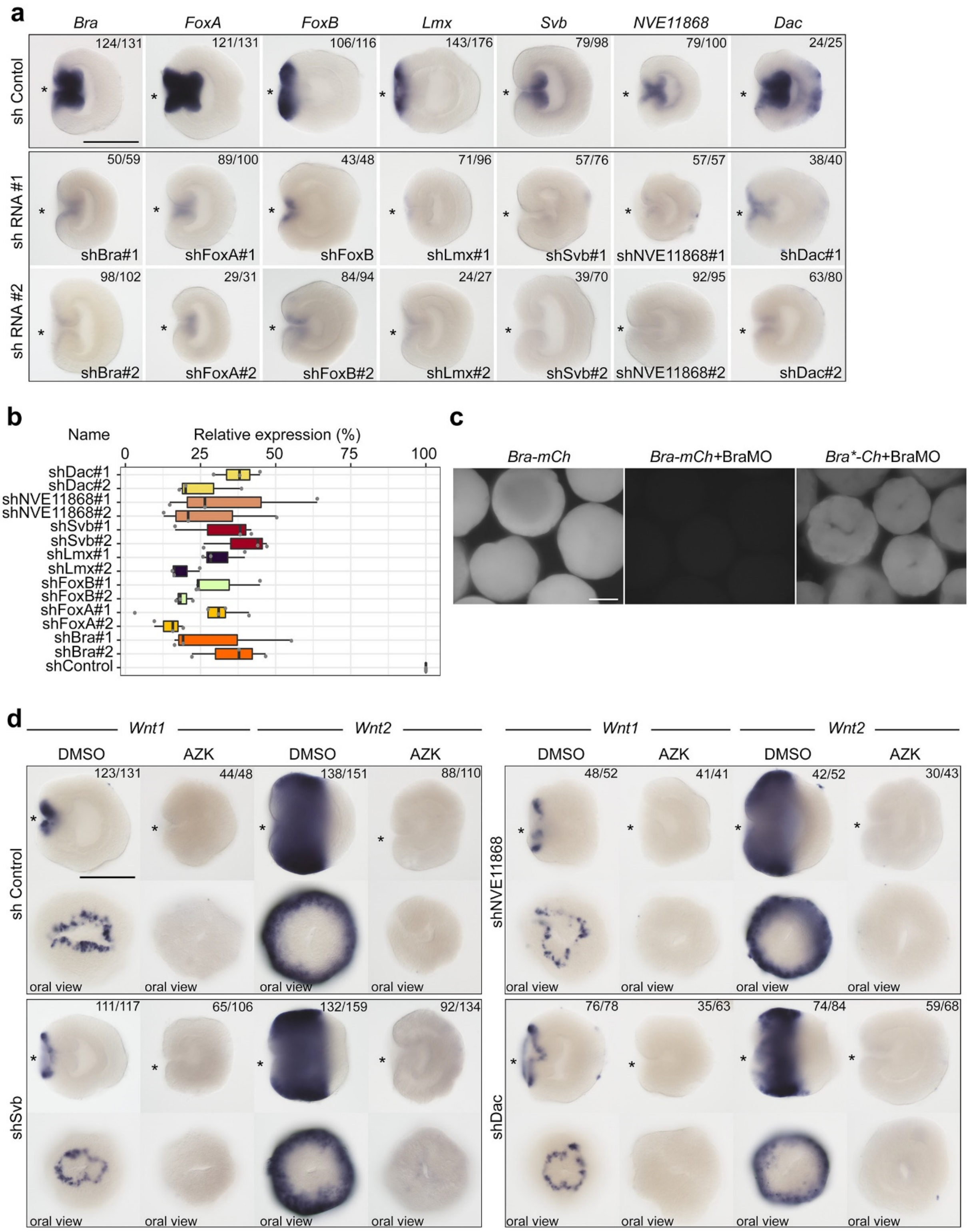

Supplementary Fig. 3: Testing the efficiency of the shRNAs and morpholino, and identification of the three candidates not fulfilling the last repressor X criterion. For each gene, two shRNAs have been selected. For *Bra*, a translation blocking morpholino was used as an alternative means of knockdown (kdn). **a,** In situ hybridization shows reduction in the staining intensity upon shRNA mediated knockdown of the repressor X candidates.

Lateral views (oral to the left) are shown. **b,** qPCR quantification of the knockdown efficiency for shRNAs used on **(a)**. For each shRNA, qPCR was performed on biological triplicates (n=3) except for shFoxA#1 (n=5). The data were normalized to GAPDH expression, and the expression is shown in percent relative to the shControl condition (set to 100%). The box represents the 25-75% interquartile range with the median indicated with the line, the whiskers represent the maximum example within 1.5x the interquartile range. Individual datum point are shown as grey dots. **c,** When co-injected, BraMO binds mRNAs containing BraMO recognition sequence fused to the mCherry coding sequence (*Bra-mCh*) and suppressed its translation. In contrast, no repression of translation is observed when BraMO is coinjected with mRNA containing a 5-mismatch recognition sequence for BraMO fused to the mCherry coding sequence (*Bra*-mCh*), (replicated twice, n>300 in each case). **d,** *Wnt1* and *Wnt2* are expressed normally in DMSO and are not de-repressed in AZK upon *Svb*, *NVE11868* and *Dac* knockdown, which eliminates these transcription factors from the list of the potential repressor X candidates. Lateral views (oral to the left) and oral views are shown. Asterisks on lateral views indicate the blastopore. On **(a)** and **(d)**, the numbers in the top right corner show the ratio of embryos displaying the phenotype shown on the image to the total number of embryos treated and stained as indicated on the figure. Scale bars 100 µm.

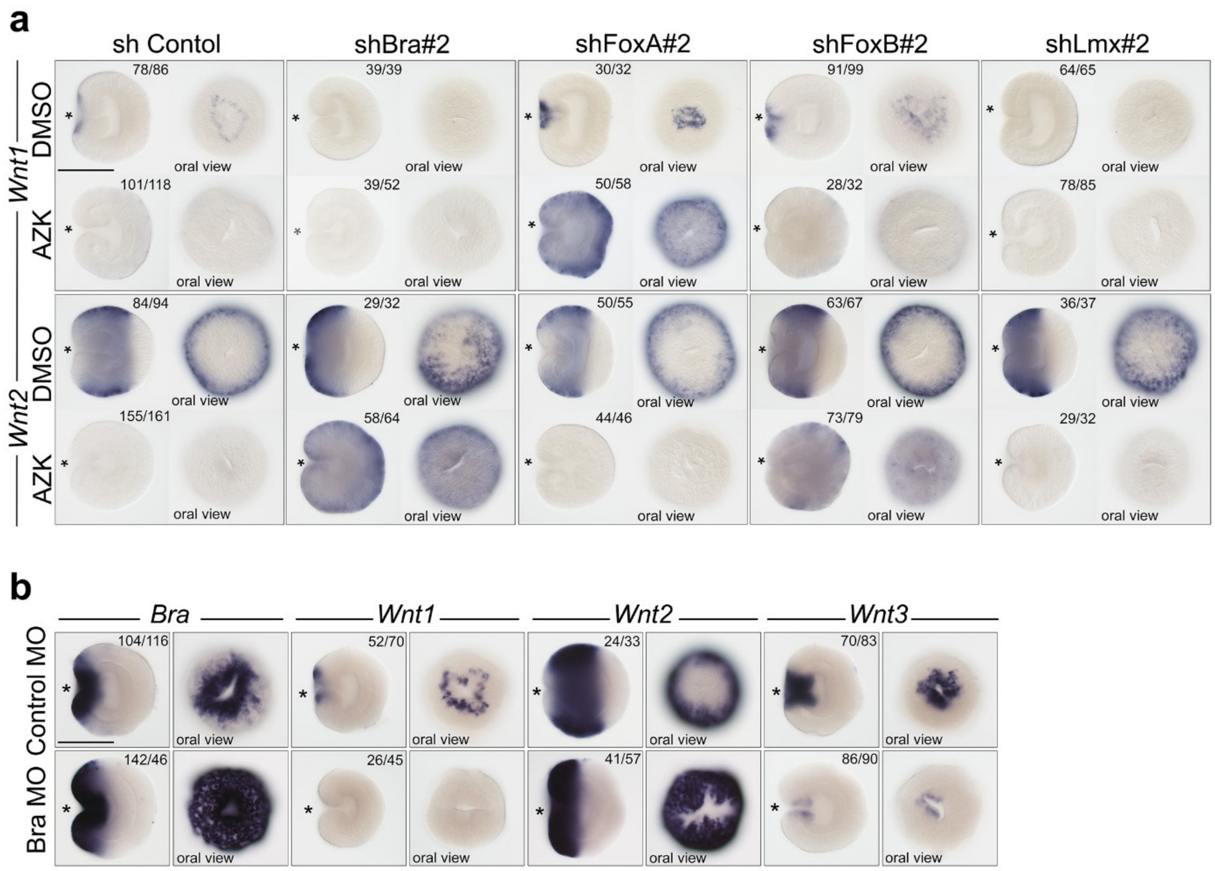

Supplementary Fig. 4: Effects of second shRNAs and of the Bra morpholino. a, The effects of the second shRNAs against repressor X candidates on Wnt1 and Wnt2 expression are similar to the effects of the first shRNA. Compare to Fig. 3 in the main text. **b,** Injection of BraMO has a similar effect on *Wnt1*, *Wnt2* and *Wnt3* expression as shBra. In contrast, *Bra* expression is clearly upregulated upon BraMO injection suggesting a negative feedback loop. The numbers in the top right corner show the ratio of embryos displaying the phenotype shown on the image to the total number of embryos treated and stained as indicated on the figure. Scale bars 100 µm.

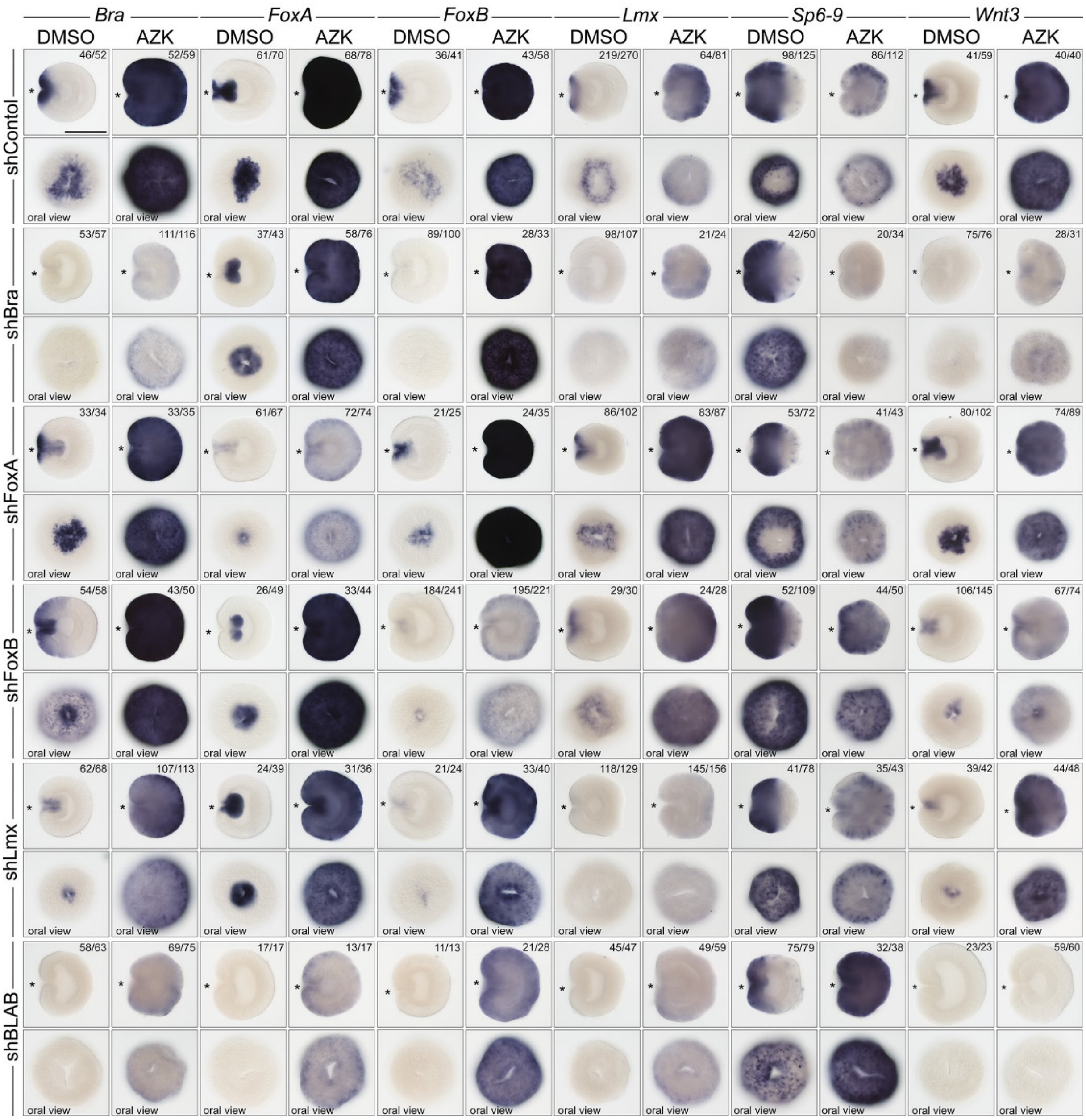

Supplementary Fig. 5: Effects of the individual knockdowns and of the simultaneous knockdown of all four repressor X candidates on their own expression, and on the expression of *Sp6-9* and *Wnt3*. The same shRNAs as on Fig. 3 are used. Lateral views (oral to the left) and oral views are shown. Asterisks on lateral views indicate the blastopore. The numbers in the top right corner show the ratio of embryos displaying the phenotype shown on the image to the total number of embryos treated and stained as indicated on the figure. Scale bar 100 µm. For interpretation, see main text and Supplementary Results and Discussion 1.

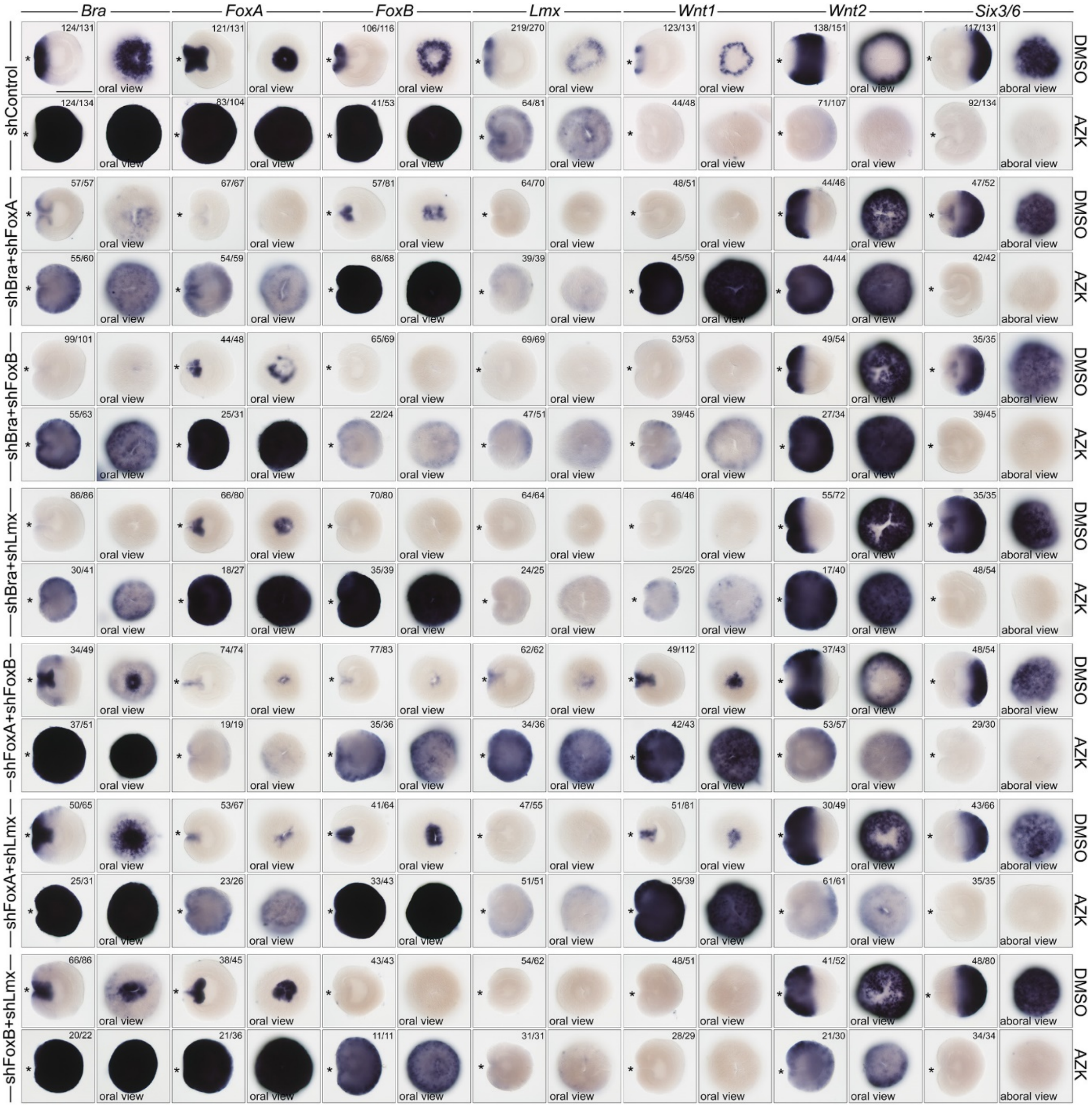

Supplementary Fig. 6: Effects of the double knockdowns of all possible combinations of the four repressor X candidates on their own expression and on the expression of *Wnt1*, *Wnt2*, and of the aboral marker *Six3/6*.

Lateral views (oral to the left) and oral views are shown. For interpretation, see main text and Supplementary Results and Discussion 1. Note oral expansion of Six3/6 upon knockdowns with shRNA combinations containing *Lmx* and, especially, *Bra*. *Bra* knockdown also leads to the expression of *Six3/6* at the bottom of the pharynx. The numbers in the top right corner show the ratio of embryos displaying the phenotype shown on the image to the total number of embryos treated and stained as indicated on the figure. Scale bar 100 µm.

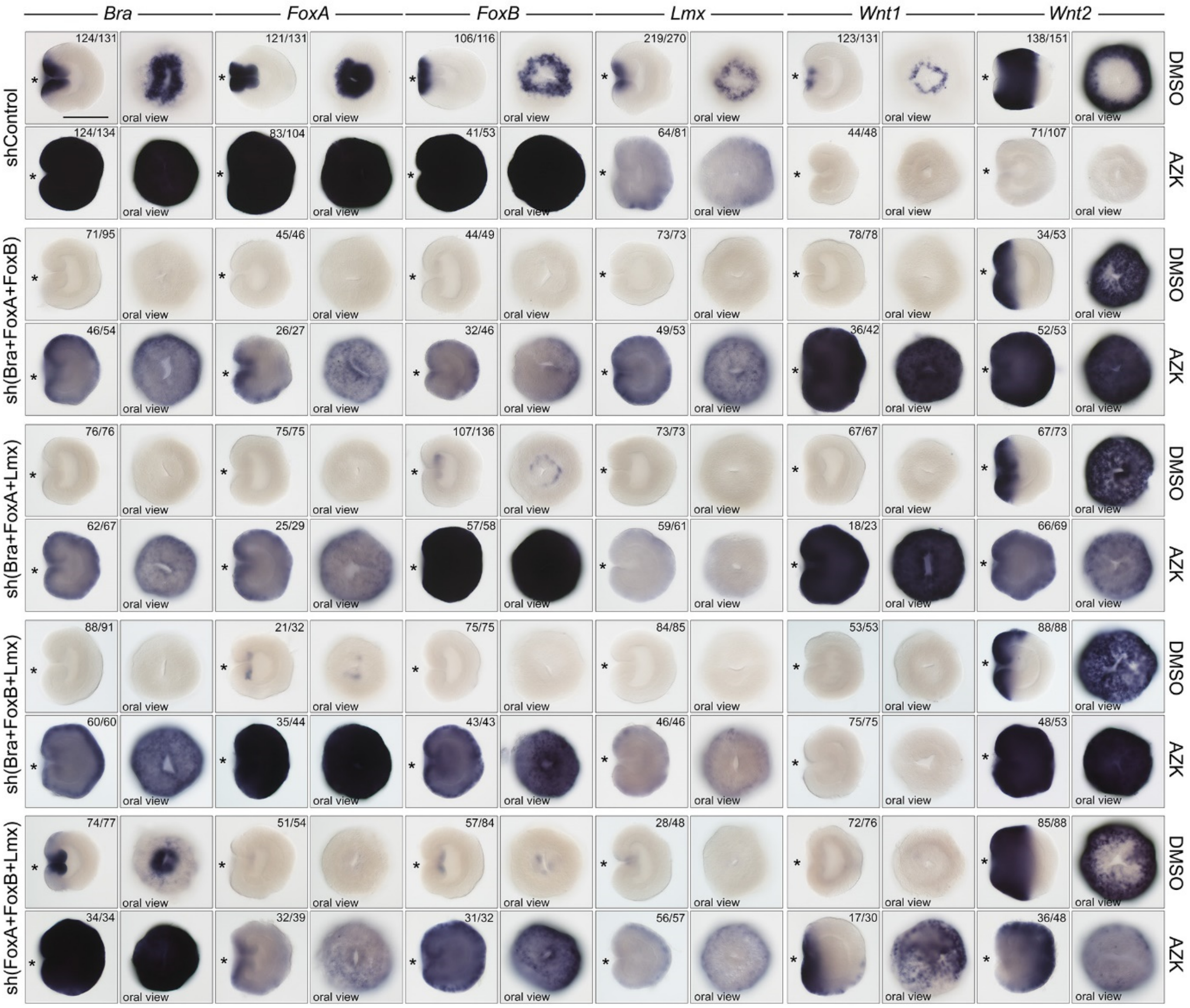

Supplementary Fig. 7: Effects of the tripple knockdowns of all possible combinations of the four repressor X candidates on their own expression and on the expression of *Wnt1* and *Wnt2*, and the results of the blastopore lip transplantation experiments. Effects of the triple knockdowns of all possible combinations of the four repressor X candidates on their own expression and on the expression of *Wnt1*, and *Wnt2*. Lateral views (oral to the left) and oral views are shown. The numbers in the top right corner show the ratio of embryos displaying the phenotype shown on the image to the total number of embryos treated and stained as indicated on the figure. Scale bar 100 µm. For interpretation, see main text and Supplementary Results and Discussion 1.

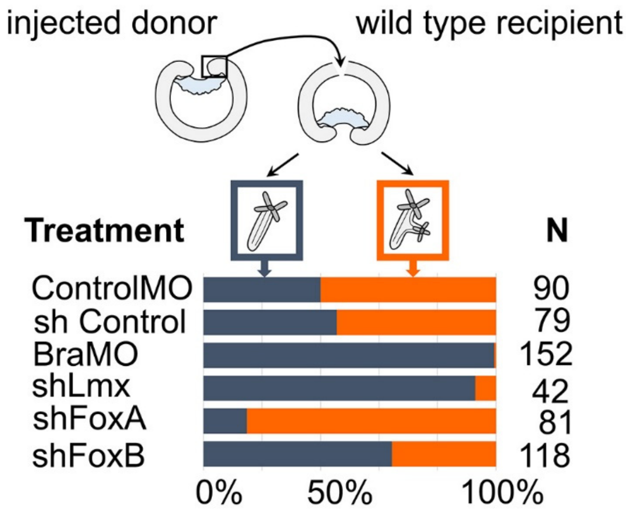

Supplementary Fig. 8: Effect of the knockdown of repressor X candidates on the inductive capacity of the blastopore lip. The ectopic axis induction capacity of the blastopore lip fragment increases drastically if *FoxA* is knocked down in the donor and sinks if *Bra* or *Lmx* expression in the donor is suppressed.

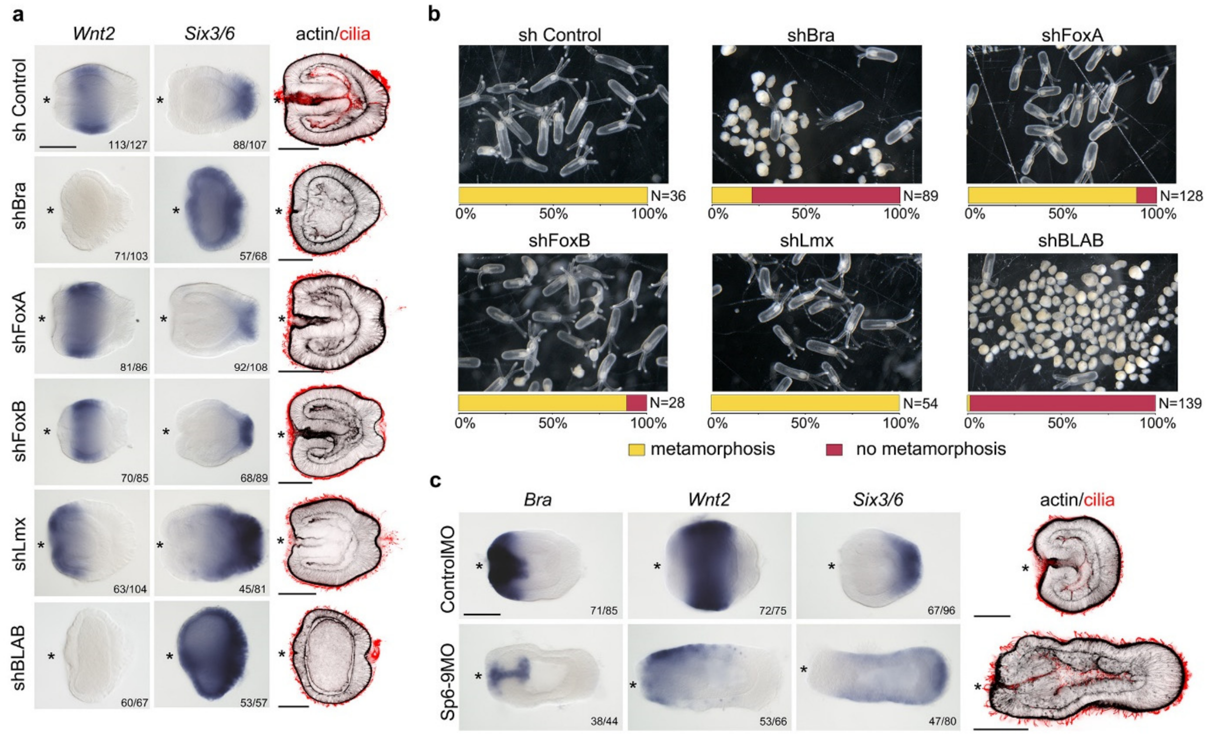

Supplementary Fig. 9: Effect of the Bra, FoxA, FoxB, Lmx and Sp6-9 knockdown on later development. a, *Wnt2* and *Six3/6* expression and morphology (F-actin is stained with phalloidin, cilia are stained with anti-acetylated Tubulin antibody) of 4 day old knockdown embryos. shBra and especially shBLAB embryos are strongly aboralized with the oral and the midbody domain as well as mouth and pharynx disappearing. shLmx embryos are mildly aboralized with the *Wnt2* expression shifted orally and *Six3/6* expression domain expanded but with no obvious effect on the morphology. shFoxA and shFoxB embryos appear normal. **b,** The proportion of metamorphosis by 10 days post fertilization. shFoxA, shFoxB and shLmx embryos are able to compensate for the effect of the knockdown and metamorphose normally. In shBra (19/89) and especially in shBLAB (2/139), metamorphosis is impaired. **c,** *Bra*, *Wnt2* and *Six3/6* expression and morphology of the 3 day old Sp6-9 knockdown embryos. The embryos are elongated and severely aboralized. On **(a)** and **(c)**, the numbers in the bottom right corner show the ratio of embryos displaying the phenotype shown on the image to the total number of embryos treated and stained as indicated on the figure. Scale bars 100 μm.

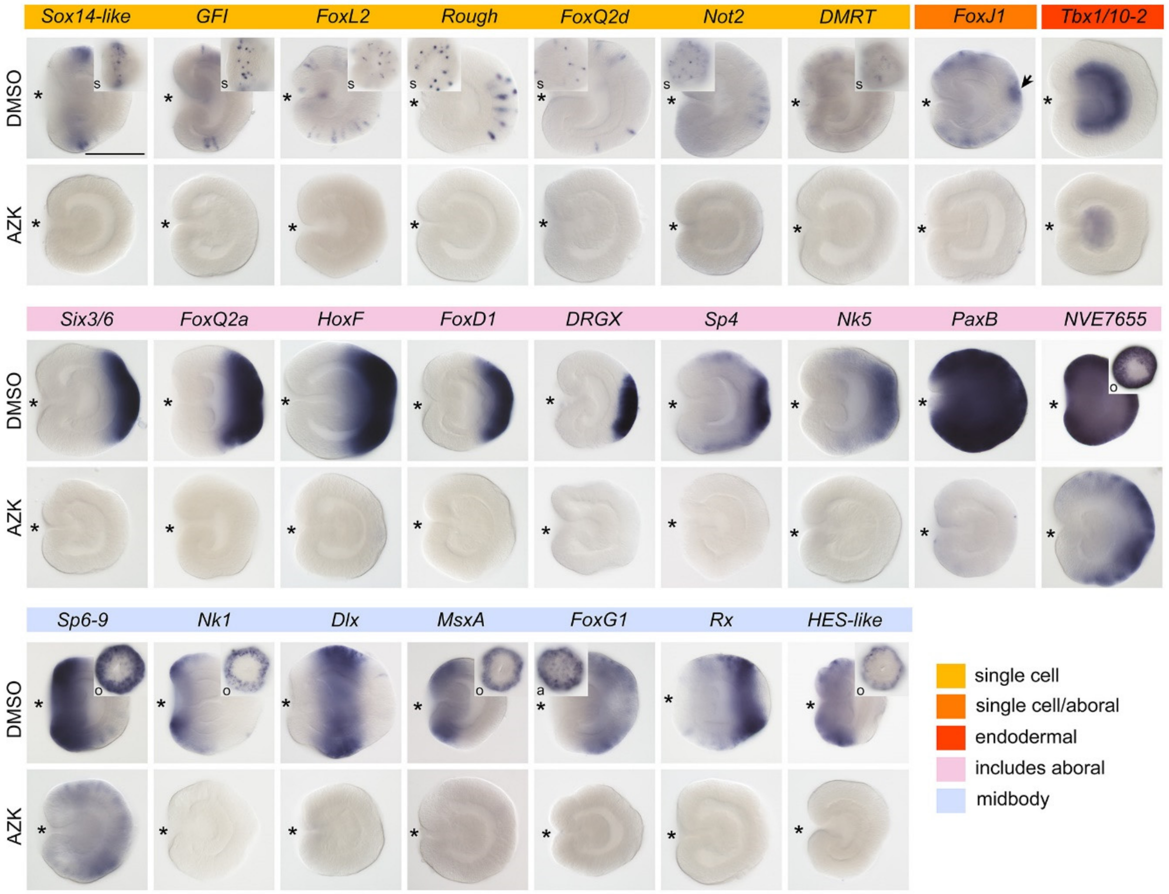

Supplementary Fig. 10: Expression of 25 transcriptional repressor Y candidates. All 25 are downregulated in AZK. Different expression groups are color-coded. Scale bar 100 µm.

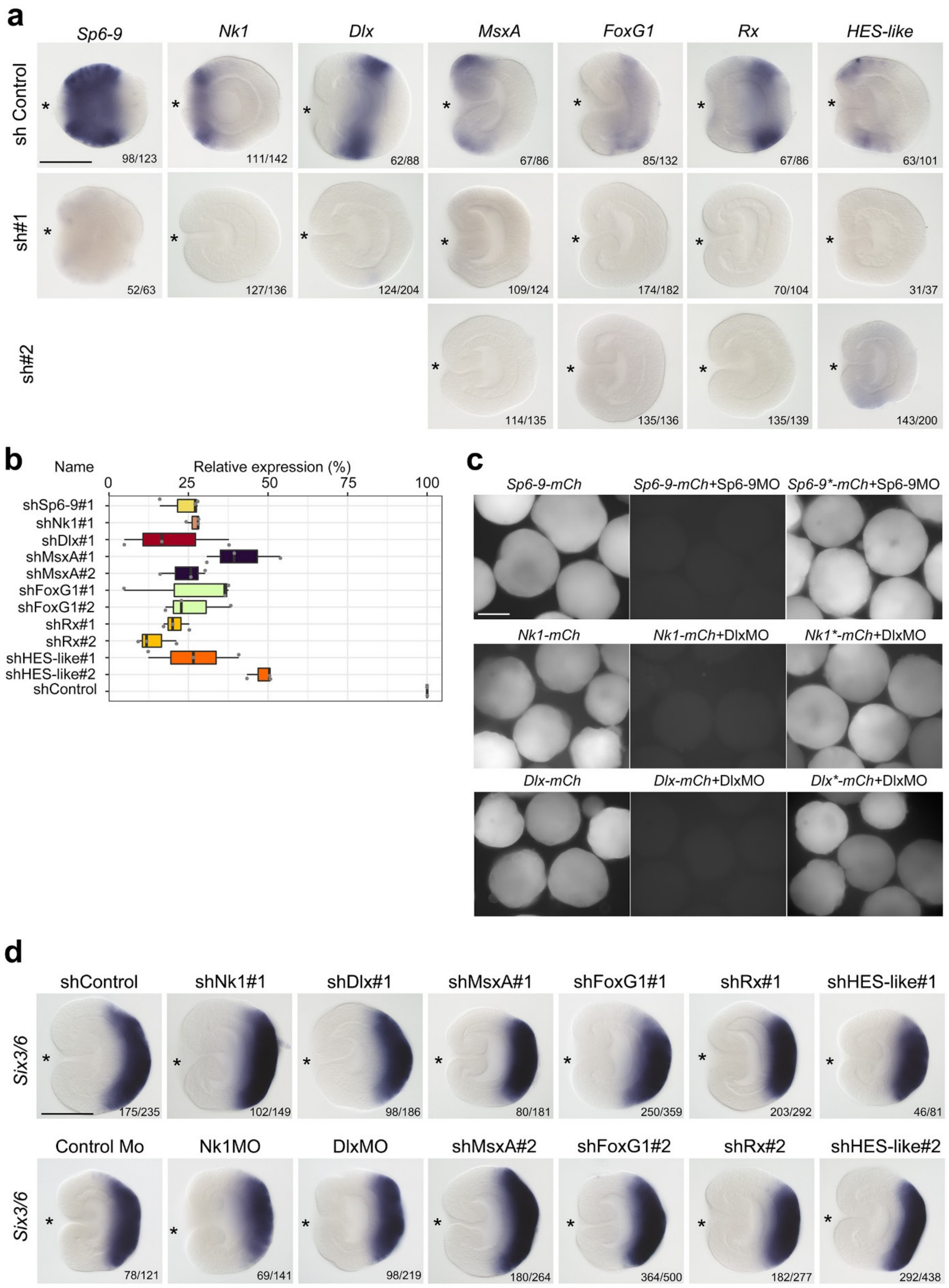

Supplementary Fig. 11: Testing the efficiency of the shRNAs and morpholinos, and identification of the six candidates not fulfilling the last repressor Y criterion. For each gene except for *Sp6-9*, *Nk1* and *Dlx*, two shRNAs have been selected. For *Sp6-9*, *Nk1* and *Dlx*, translation blocking morpholinos were used as an alternative means of knockdown (kdn)

since no second functional shRNA could be found. **a,** In situ hybridization shows reduction in the staining intensity upon shRNA mediated knockdown of the repressor Y candidates. Lateral views (oral to the left) are shown. **b,** qPCR quantification of the knockdown efficiency for shRNAs used on **(a)**. For each shRNA, qPCR was performed on biological triplicates (n=3). The data were normalized to GAPDH expression, and the expression is shown in percent relative to the shControl condition (set to 100%). The box represents the 25-75% interquartile range with the median indicated with the line, the whiskers represent the maximum example within 1.5x the interquartile range. Individual datum point are shown as grey dots. **c,** When co-injected with mRNAs containing their respective recognition sequences fused to the mCherry coding sequence (*Sp6-9-mCh, Nk1-mCh, Dlx-mCh*), Sp6-9MO, Nk1MO and DlxMO bind and suppress their translation. In contrast, no repression of translation is observed when Sp6-9MO, Nk1MO and DlxMO are coinjected with mRNAs containing their 5-mismatch recognition sequences fused to the mCherry coding sequence (*Sp6-9*-mCh, Nk1*-mCh, Dlx*-mCh*). Replicated twice, n>300 in each case. **d,** *Six3/6* is expressed normally upon shRNA- or MO-mediated knockdown of six out of seven repressor Y candidates . Lateral views (oral to the left) and oral views are shown. On **(a)** and **(c)**, the numbers in the bottom right corner show the ratio of embryos displaying the phenotype shown on the image to the total number of embryos treated and stained as indicated on the figure. Asterisks on lateral views indicate the blastopore. Scale bars 100 µm.

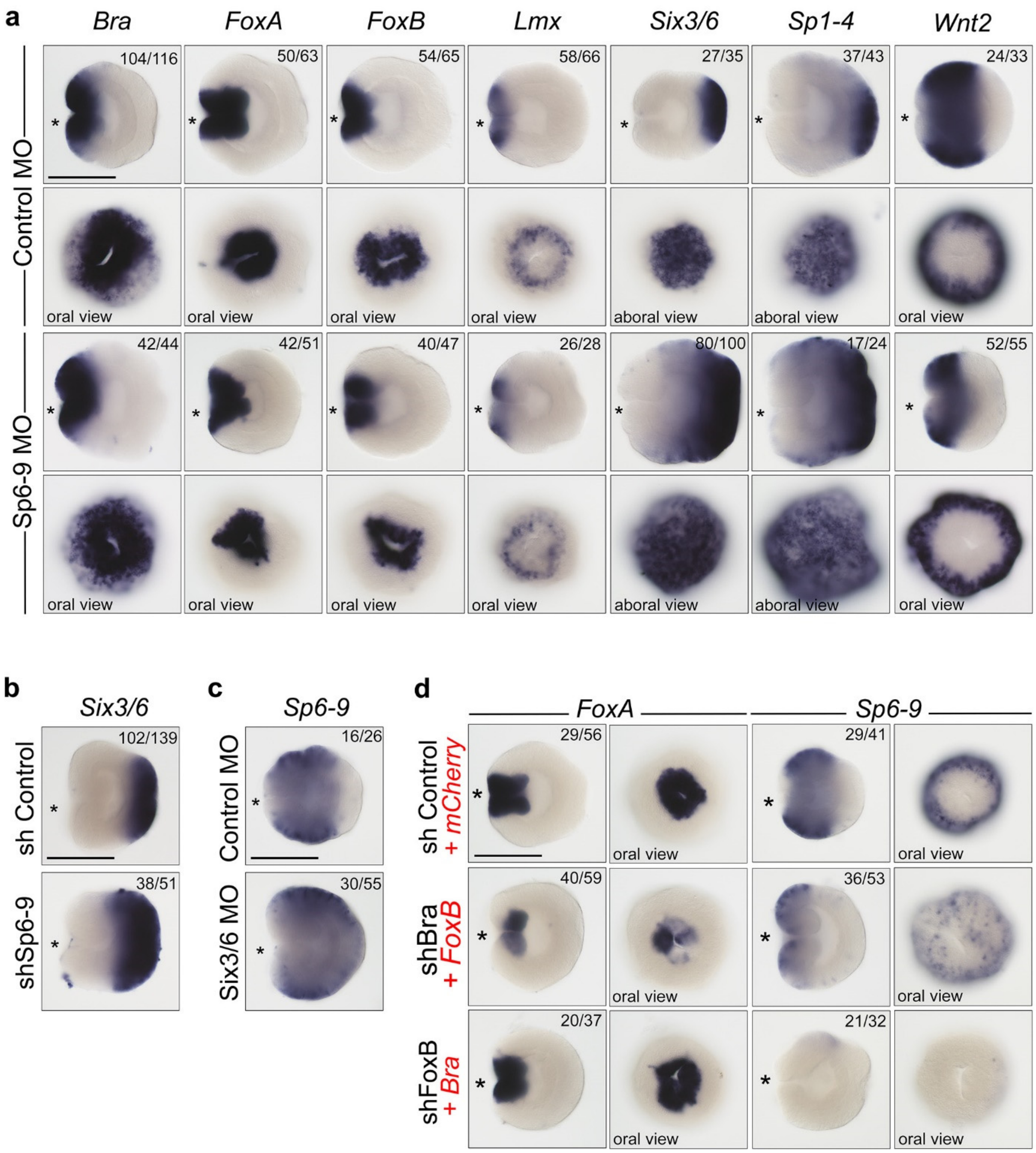

Supplementary Fig. 12: Functional analysis of *Sp6-9* and rescue experiments with *Bra* and *FoxB*. a, Sp6-9MO does not affect *Bra*, *FoxA*, *FoxB* and *Lmx* expression. *Wnt2* ring becomes narrower, while *Six3/6* and *Sp1-4* expression domains expand orally. **b,** shSp6-9 affects *Six3/6* expression in the same way as the Sp6-9MO. **c,** Six3/6MO injection leads to aboral expansion of *Sp6-9*. **d,** Effects of the co-injection of the indicated shRNA (black letters) and mRNA (red letters). Ubiquitous *Bra* expression compensates for the lack of FoxB and rescues *FoxA* in its normal domain without causing ectopic overexpression. In contrast, *FoxB* mRNA does not rescue the shBra effect on *FoxA* expression. *Bra* overexpression abolishes *Sp6-9* irrespective of the lack of FoxB, while *FoxB* overexpression makes the shBra

effect on *Sp6-9* milder without alleviating it completely (weaker *Sp6-9* expression with a milder oral expansion in comparison to shBra alone). Lateral views (oral to the left) and oral views are shown. See Supplementary Fig. 5 for the shBra and shFoxB knockdown phenotypes. The numbers in the top right corner show the ratio of embryos displaying the phenotype shown on the image to the total number of embryos treated and stained as indicated on the figure. Scale bars 100 µm.

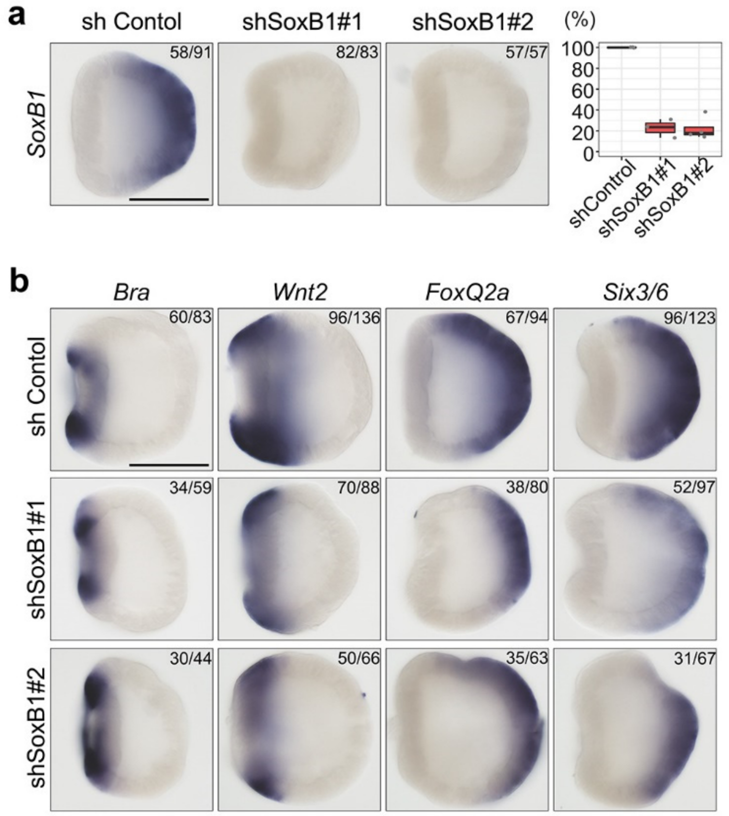

Supplementary Fig. 13: *SoxB1* is repressed in the midbody domain and does not seem to be the critical regulator of *Six3/6* and FoxQ2a expression. a, *SoxB1* is efficiently knocked down by two independent shRNAs, as demonstrated by in situ hybridization and qPCR (n=3; the box represents the 25-75% interquartile range with the median indicated with the line, the whiskers represent the maximum example within 1.5x the interquartile range. Individual datum point are shown as grey dots). **b,** *SoxB1* knockdown does not affect the expression of oral and midbody markers *Bra* and *Wnt2*, and appears to slightly reduce the expression of the aboral markers *Six3/6* and *FoxQ2a* at the 18 hpf pregastrula stage. The numbers in the top right corner show the ratio of embryos displaying the phenotype shown on the image to the total number of embryos treated and stained as indicated on the figure. Scale bars 100 µm.

Supplementary Results and Discussion

1. Topology of the gene regulatory network

In order to understand the genetic interactions between the four factor X candidates we analyzed their expression in individual, double, triple and quadruple knockdowns (Supplementary Fig. 5-7). While identification of the exact topology of this network requires stage-by-stage ChIP data for all the transcription factors from the onset of their expression until late gastrula, we can suggest a possible topology based on genetic interactions. Upon shRNA mediated knockdown of *Bra*, *FoxB* and *Lmx* become abolished. *FoxA* expression is confined to the bottom of the forming pharynx, where *Bra* is not expressed, suggesting that Bra activates the expression of these three genes in the area where they are normally co-expressed with *Bra* (Supplementary Fig. 5). The effect of *Bra* knockdown on *Bra* expression is more complex: while shRNA-mediated knockdown reduces the amount of *Bra* mRNA (Supplementary Fig. 5), the translation blocking morpholino-mediated knockdown of *Bra* upregulates the expression of *Bra* gene (Supplementary Fig. 4b). The most likely explanation for this is that Bra protein may act as a transcriptional repressor of the *Bra* gene. In contrast, the effects of the shRNA-mediated and morpholino-mediated knockdown of *Bra* on *Wnt1*, *Wnt2*, and *Wnt3* expression are essentially the same (Fig. 3, Supplementary Figs. 4-5). While *Wnt2* clearly appears to be de-repressed by *Bra* knockdown, the expression *Wnt1* and *Wnt3* becomes abolished in DMSO and in AZK (Fig. 3, Supplementary Fig. 5). Interestingly, *Wnt1* is expressed in AZK in quadruple knockdowns (Fig. 3) and in triple knockdowns, whenever shFoxA is used (Supplementary Fig. 7a). Thus, the regulation of *Wnt1* by β-catenin may still be direct. *Wnt3*, in contrast, is abolished in AZK in quadruple knockdowns (Supplementary Fig. 5b). This suggests that *Wnt3* is either regulated by β-catenin indirectly via Bra and FoxB, or that a yet unidentified "window" transcriptional repressor normally preventing aboral expansion of the *FoxA*/*Wnt1*/*Wnt3* boundary (Fig. 2) becomes de-repressed in AZK upon quadruple knockdown and suppresses *Wnt3* (just like *Wnt2* becomes de-repressed in shBLAB and expands orally).

According to double and triple knockdowns followed by AZK treatment, *Bra, FoxB*, *Lmx* and *FoxA* are likely to be direct β-catenin targets (Supplementary Fig. 6-7). Upon individual *FoxB* shRNA knockdown, in DMSO, the ring of *Wnt1* concentrates around the blastopore opening and appears weaker, and in AZK, *Wnt1* expression expands aborally but remains weak and absent in blastopore lips (Fig. 3). *Wnt2* expression in *FoxB* knockdown is slightly expanded

orally in DMSO and globally in AZK, but also remains weak (Fig. 3). This is strikingly similar to the effects we documented in wild type embryos treated with lower concentrations of AZK[2]. The effect of its knockdown on the expression of the *Wnt* genes suggests that FoxB might act as an enhancer of Bra and FoxA activity. The role of *Lmx* is also not fully clear, since the effect of its knockdown on *Wnt1* and *Wnt2* seems to be weaker but similar to the effect of the *Bra* knockdown (Fig. 3), suggesting that Bra and Lmx proteins might cooperate in regulating the same targets. The only observable difference is that *Lmx* is not co-expressed with *Wnt3* (Fig. 2), which is localized to the *FoxA/Bra* co-expression domain, and *Lmx* knockdown does not abolish *Wnt3* expression (Supplementary Fig. 5). The role of Lmx and FoxB appears to be in supporting the expression and function of *Bra* in their respective co-expression domains (Fig. 2, Supplementary Fig. 5-7, see also main text), while strong FoxA appears to suppress *Bra* expression in the absence of FoxB (i.e. at the bottom of the pharynx, Supplementary Fig. 5). Similar expression of *Bra* upon single knockdown of *FoxB* (Supplementary Fig. 5), double knockdown of *FoxB*+*Lmx* or *FoxB*+*FoxA* (Supplementary Fig. 6), and triple knockdown of *FoxB*+*FoxA*+*Lmx* (Supplementary Fig. 7) points towards the critical role of FoxB in maintaining the normal domain of strong *Bra* expression. One yet unclear effect is the nearly normal expression of *Bra* (normal domain plus bottom of the pharynx) and upregulation of *FoxB* upon double knockdown of *Lmx* and *FoxA* (Supplementary Fig. 6), although single knockdowns of these genes reduced *Bra* expression restricting it to the bottom of the pharynx (Supplementary Fig. 5). In spite of the effects of the *FoxB* knockdown and the *Bra* knockdown on *FoxA* expression being highly similar (expression is confined to the bottom of the pharynx, Supplementary Fig. 5), overexpression of *FoxB* in the shBra background does not rescue the *FoxA* phenotype (Supplementary Fig. 12d). In contrast, overexpression of *Bra* mRNA in the shFoxB background completely rescues the shFoxB effect on *FoxA* without inducing *FoxA* expression outside of its normal domain (Supplementary Fig. 12d). The lack of ectopic expression of *FoxA* upon *Bra* overexpression suggests that the genes encoding transcription factors repressing *FoxA* aborally are not under Bra control. Identical effects of *Bra*, *FoxB* and *Lmx* knockdowns on the expression of the midbody marker *Sp6-9* (Supplementary Fig. 5) suggest that these factors might co-operate in preventing the oral expansion of the expression domain of this gene. However, Bra is clearly the key player in this inhibition: co-injection of shBra with *FoxB* mRNA does not fully suppress *Sp6-9* expression, although *Sp6-9* appears to be much weaker than in control, and its oral expansion appears to be less pronounced in comparison to the shBra alone (compare Supplementary Fig. 5 and Supplementary Fig. 12d). In contrast, co-

injection of shFoxB with *Bra* mRNA drastically reduces *Sp6-9* expression (Supplementary Fig. 12d). Notably, *Sp6-9* knockdown does not cause aboral expansion of *Bra*, *FoxA*, *FoxB* and *Lmx* (Supplementary Fig. 12a), suggesting that they may be suppressed by the yet unidentified midbody genes. Similarly, it is unclear what prevents aboral expansion of the other oral markers expressed in concentric rings, e.g. *Wnt1*, *Wnt3*, *Wnt4* and *WntA* (see Fig. 2). In contrast, Sp6-9 clearly prevents oral expansion of the aborally expressed *Six3/6, Sp1-4* and *SoxB1* (Figs. 4c and 5c; Supplementary Fig. 12a-b). Conversely, *Six3/6* knockdown results in the aboral expansion of *Sp6-9* (although the penetrance is not too high at 55%, N=55) (Supplementary Fig. 12c). Since the suppression of *Sp6-9* alone does not result in the aboral expansion of *Bra*, it is clear that some yet unidentified factors are involved in preventing aboral expansion of the oral and midbody genes. The deduced topology of the genetic interactions in this GRN is summarized in the Fig. 6a.

2. Knockdown of the four oral TFs affects de novo axis formation but not the normal gastrulation

Our analysis of the effects of the knockdowns of the four transcription factors defining oral identity in *Nematostella* provided another highly surprising result. Although gastrulation in *Nematostella* is abolished by β-catenin morpholino[3], and ectodermal co-expression of *Wnt1* and *Wnt3* is sufficient to induce axis and germ layer formation at any positon in the embryo [2,4], neither individual knockdowns nor the quadruple knockdown of *Bra*, *Lmx*, *FoxA* and *FoxB*, affected the process of gastrulation in any detectable way. This was unexpected, since *Bra* knockdown abolishes both *Wnt1* and *Wnt3*, and *Lmx* knockdown abolishes *Wnt1* (Fig. 3; Supplementary Fig. 5). In order to address this discrepancy, we tested whether the expression of our four candidate transcription factors affects ectopic axis induction by blastopore lip transplantation (Supplementary Fig. 8). We predicted that if any of these molecules were necessary for axis induction, their loss from the donor blastopore lip tissue would abolish its inductive potential. Indeed, the knockdowns of *Bra* and *Lmx* nearly abolished the inductive capacity of the blastopore lip when compared to the transplantations from Control MO or shControl injected embryos (Z-test, $p<1e-5$ in both cases), and *FoxB* knockdown significantly reduced it (Z-test, $p<0.01$). This latter effect might be due to the reduced *Wnt1* and *Wnt3* expression in shFoxB embryos (Fig. 3, Supplementary Fig. 5). In contrast, the knockdown of *FoxA*, which appears to be the transcriptional repressor of *Wnt1*, strongly increased induction efficiency (Z-test, $p<1e-5$). This suggests that all these factors affect the ectopic axis and germ

layer formation, while normal gastrulation in the embryo relies primarily on maternally deposited determinants.

3. Effects of the *Bra*, *FoxA*, *FoxB*, *Lmx* and *Sp6-9* knockdown on later development

We then asked what the effects of the knockdowns of the four oral TFs were in terms of later development. Since most of the orally expressed marker genes appeared to be controlled by Bra at the gastrula stage, we focused on analyzing the expression of the midbody and the aboral ectoderm markers *Wnt2*, and *Six3/6*, general morphology and percentage of metamorphosis. Among the shBra, shFoxA, shFoxB, shLmx, and shBLAB, only shBra and shBLAB showed a strong aboralization phenotype by 4 dpf (late planula larva) (Supplementary Fig. 9a). Marker gene expression upon individual knockdown of other three genes appeared either normal in shFoxA and shFoxB or aboralized in shLmx, with *Wnt2* and *Six3/6* expression expanding orally in more than 50% of the embryos. However, morphologically, shFoxA, shFoxB, and shLmx knockdown embryos appeared normal for their developmental stage (Supplementary Fig. 9a). The aboralization of shBLAB embryos was similar but always more pronounced than that of the shBra embryos. These embryos appeared shorter than controls, they lacked mouths and pharynges, and their endoderm partitioning was missing or heavily disrupted. At the molecular level, the midbody marker *Wnt2* was abolished, which is different from the situation in the gastrula, and may be due to the fact that Bra is a positive regulator of the oral Wnt genes (e.g. Wnt1 and Wnt3, see Fig. 3, Supplementary Fig. 5). Consistent with the loss of the oral and the midbody domain, the aboral marker *Six3/6* was ubiquitously expressed (Supplementary Fig. 9a). This is reflected by the percentage of the knockdown embryos capable of undergoing metamorphosis (scored at 10 dpf). In contrast to shControl injected embryos (100% metamorphosis, N=36), shFoxA injected embryos (89% metamorphosis, N=128), shFoxB injected embryos (89% metamorphosis, N = 28) and shLmx injected embryos (100% metamorphosis, N=54), only 21% (N=89) of the shBra injected and 1% (N=139) of the shBLAB injected embryos underwent metamorphosis and formed primary polyps (Supplementary Fig. 9b). Normal gastrulation followed by the loss of the pharynx and the disappearance of the *Wnt2* transcript at later developmental stages is consistent with the previously reported results of the CRISPR/Cas9-mediated excision of *Bra* locus by simultaneous use of 5 non-overlapping gRNAs in F0[5]. The fact that shBra and shBLAB embryos survived until 10 dpf and had an

uncompartmentalized but otherwise more or less normally looking endoderm (Supplementary Fig. 9a) suggests that the death of the embryos by day 4 reported previously[5] may have been caused by some unaccounted for off-target action of the gRNAs rather than by the lack of pharynges, as it was suggested.

We then tested late developmental effects of the *Sp6-9* knockdown on the morphology and expression of the oral ectoderm marker *Bra*, midbody marker *Wnt2* and aboral marker *Six3/6*. By 3 dpf, the injection of the Sp6-9 morpholino resulted in the formation of worm-like embryos with a shortened oral and strongly elongated aboral domain and non-compartmentalized endoderm. The expression of the oral marker *Bra* and the midbody marker *Wnt2* was reduced, and *Six3/6* was significantly expanded orally (Supplementary Fig. 9c). Sp6-9 morphant embryos do not undergo metamorphosis and die at some point between 4 and 7 dpf.

Supplementary Table 1: Transcriptional repressor X candidates

GeneID	Curated ID	Fold change				Expression
		3d APCmut vs. WT	3dAZKc vs. DMSO	3dAZKwo vs. DMSO	1dAZK vs. DMSO	
NVE21786*	*EIF3C*	1.62	1.92	1.50	1.34	not assayed
NVE12602*	*ORC1*	1.66	1.83	1.64	1.58	not assayed
NVE12977	*MsxC*	2.08	5.78	4.35	3.99	not expressed
NVE14550	*Unc4*	17.79	19.23	5.38	2.87	single cells
NVE20732	*AshC*	13.35	13.70	4.63	3.92	single cells
**NVE3568**	***Brachyury***	**8.98**	**9.52**	**2.97**	**3.34**	**oral ectoderm**
**NVE20630**	***FoxA***	**10.05**	**12.05**	**5.85**	**5.45**	**oral ectoderm**
**NVE26195**	***FoxB***	**45.00**	**40.00**	**5.03**	**4.84**	**oral ectoderm**
**NVE16579**	***Lmx***	**5.85**	**4.67**	**2.59**	**3.22**	**oral ectoderm**
**NVE13527**	***Shavenbaby***	**3.04**	**3.57**	**4.15**	**2.08**	**oral ectoderm**
**NVE24711**	***Dachshund***	**4.52**	**4.52**	**4.93**	**1.99**	**oral ectoderm**
**NVE11868**	***Zn finger protein***	**12.55**	**6.99**	**5.15**	**3.27**	**oral ectoderm**

* NVE21786 and NVE12602 are greyed out as metabolic enzymes falsely automatically annotated as transcription factors

Supplementary Table 2: Transcriptional repressor Y candidates

GeneID	Curated ID	Fold change				Expression
		3d APCmut vs. WT	3dAZKc vs. DMSO	3dAZKwo vs. DMSO	1dAZK vs. DMSO	
NVE15777	*Sox14-like*	0.05	0.04	0.02	0.34	single cells
NVE16639	*GFI*	0.37	0.46	0.39	0.23	single cells
NVE1324	*FoxL2*	0.06	0.34	0.41	0.57	single cells
NVE21292	*Rough*	0.10	0.24	0.15	0.35	single cells
NVE17371	*FoxQ2d*	0.03	0.02	0.02	0.15	single cells
NVE4967	*Not2*	0.27	0.25	0.14	0.27	single cells
NVE4006	*DMRT*	0.29	0.39	0.08	0.57	single cells
NVE14608	*FoxJ1*	0.12	0.18	0.30	0.30	single cells/aboral
NVE8569	*TBX1/10-2*	0.29	0.37	0.17	0.26	endodermal
NVE12346	*Six3/6*	0.06	0.04	0.03	0.14	includes aboral
NVE14268	*FoxQ2a*	0.04	0.02	0.03	0.12	includes aboral
NVE16373	*HoxF/Anthox1*	0.04	0.03	0.02	0.15	includes aboral
NVE21434	*FoxD1*	0.09	0.03	0.03	0.04	includes aboral
NVE14554	*DRGX*	0.11	0.17	0.08	0.08	includes aboral
NVE21395	*Sp1-4*	0.26	0.12	0.46	0.28	includes aboral
NVE20898	*Nk5*	0.08	0.08	0.40	0.23	includes aboral
NVE5430	*PaxB*	0.17	0.27	0.15	0.54	includes aboral
NVE7655*	*unknown*	0.11	0.11	0.11	0.44	includes aboral
**no model**	***Sp6-9***	**0.08**	**0.31**	**0.24**	**0.60**	**midbody**
**NVE20899**	***Nk1***	**0.07**	**0.28**	**0.07**	**0.35**	**midbody**
**NVE8363**	***Dlx***	**0.25**	**0.25**	**0.15**	**0.37**	**midbody**
**NVE20892**	***MsxA***	**0.09**	**0.19**	**0.10**	**0.44**	**midbody**
**NVE6876**	***FoxG1***	**0.04**	**0.04**	**0.03**	**0.13**	**midbody**
**NVE21445**	***Rx***	**0.04**	**0.12**	**0.25**	**0.31**	**midbody**
**NVE14243**	***HES-like***	**0.10**	**0.20**	**0.26**	**0.41**	**midbody**

* NVE7655 encodes a long uncharacterized protein, which is probably falsely automatically annotated as a transcription factor

Supplementary Table 3: Short hairpin RNA targets

Name	Targeted sequence
shControl	GCGAGTTCTTCTACAAGGTGA
shBra#2	GAAGAGATCACGAGTCTAA
shBra	GAATCGCACTCAGCTTACT
shFoxA#2	GCAGGTATGCCCATGAATA
shFoxA	GCTCAAGAAATCCAAGGACAA
shFoxB#2	GAGAAGACGAGGATGAACT
shFoxB	GATTCCCTCTCTTCCTACA
shLmx#2	GTGTCACATTCTCCGTACAT
shLmx	GCTTGAGTGTAAGAGTGGT
shSvb#2	GAACCTTAGATCGGAGAGA
shSvb	GCTCCGAGAAGAGAATGTT
shNVE11868#2	GAATGACCTTGAGTGAAGA
shNVE11868	GGAGAGAGAGGTAACTGAT
shDac#2	GCAGAACAGCGAGTAACAA
shDac	GACTCTACTGAGGAACATA
shSp6-9	GCTTGAGGGATCGACTTCA
shSoxB1	GCAGCACAGTCCTTTAATA
shSoxB1#2	GGATCCTACTCGAACATGT
shNk1#1	GCAAGGACTGCTTTCACAT
shDlx#1	GCTTGTCACCGCCTGTATT
shMsxA#1	GCAGTACGACGGAAGATTT
shMsxA#2	GGACTACAAAGCAACTTCT
shFoxG1#1	GAAAGCGCAGAGGAAAGAA
shFoxG1#2	GAGGAGAAGAGATTGACTT
shRx#1	GAGCTCCAACGATGGTAAA
shRx#2	GAACAAGAGCGAAAGACTA
shHES-like#1	GAGTGTGCGCTAGAAGTTA
shHES-like#2	GCTCATCAAACGAGTTCAA

See Supplementary Fig. 3b and 11b for the knockdown efficiency estimation by qPCR and in situ hybridization

Supplementary Table 4: Morpholino sequences

Name	Morpholino sequence	Reference
BraMO	TCGTCCGAGTGCATGTCCGACTATG	new
DlxMO	TCTGGTTTCATGTAATAGGGTACTG	new
Nk1MO	TCAGGCCGCAGCATTTGAAGC	new
Sp6-9MO	TCTAGTAGTTCCTGTGAGTAGACAG	new
ControlMO	GATGTGCCTAGGGTACAACAACAAT	[2]
Six3/6MO	GTACTGCCGCACTGCAAGACTTGTC	[6]
β-cateninMO	TTCTTCGACTTTAAATCCAACTTCA	[3]

See Suppl. Fig. 3c and 11c for the confirmation of the sequence-specific activity of the new MOs.

Supplementary Table 5: Primers used for Q-PCR (marked as qF and qR) and for cloning new gene fragments

GeneId	Primer name	Primer 5'->3'
NVE3568	Bra_qF	CGCACTCAGCTTACTCCCAA
	Bra_qR	AGGTCGATGACTTCGGATGC
NVE20630	FoxA_qF	GCCATGGGTATGGCAGGTAT
	FoxA_qR	TGAAGTGCATGGGGTCGTAG
NVE26195	FoxB_qF	AAACAGTTACGGCAGCGCTAA
	FoxB_qR	GGGAAAATGGTCCATGATGA
NVE16579	Lmx_qF	GACCGAAAGGGACATCAAAGAA
	Lmx_qR	GTAAGGATTGTTCGCGGTCTT
NVE13527	Shavenbaby_qF	GCGATCATGGAACAAGGAACT
	Shavenbaby_qR	AATCCACTTCCCCCTTTCCT
NVE24711	Dachshund_qF	TCCTCACCATCCACAAACTCC
	Dachshund_qR	GCAATATCAATGCCATTCACG
NVE11868	NVE11868_qF	ATACGACAAGAGGCCACGCA
	NVE11868_qR	TCCTCCTGTTCCTCGCACTT
NVE20892	MsxA_qF	ACGGAAACACAAAGCGAATC
	MsxA_qR	TGCAGAAAACTCAGCCCTTT
NVE20899	Nk1_qF	GAACGGAGTGATCGAAGAGC
	Nk1_qR	CTTGTTGGGATCGAGGATGT
NVE8363	Dlx_qF	CGCCTACTTAGGGCTGACAC
	Dlx_qR	GTCGAGGAGCCACTGTTTTC
NVE21445	Rx_qF	CTGAAGCCACAAAACATCCA
	Rx_qR	CTTGGGTTGGAGTCGTTGAT
NVE14243	HES-like_qF	CTTCCACCAGCTGTCAGTCA
	HES-like_qR	TTGAAACTGTGTGGGACCAA
NVE6876	FoxG1_qF	CCAGGCAATAAAGAGGGTGA
	FoxG1_qR	TGATAAGCGCGTTGTACGAG
no NVE model	SP6-9_qF	GCAGCCTCGTATCATCCTCC
	SP6-9_qR	GTGGTTCCCTACAGTCACCG
NVE23709	SoxB1_qF	AAGAAATGCCTAGCCCCACA
	SoxB1_qR	CTGCCGGAGGGAGATAAGTG

Supplementary Table 5 (continued)		
NVE12977	MsxC_F	CTGACCCGCTCAAGATCACT
	MsxC_R	AGTAACATCTTCCGCGCTCT
NVE14550	Unc4_F	ATGAGAGTGAGGACGAACTTC
	Unc4_R	CATTCCTCGTGCTCCTTTGC
NVE16579	Lmx_F	GACCGAAAGGGACATCAAAGAA
	Lmx_R	CACATCGAGGCTTGGCTTTC
NVE13527	Shavenbaby_F	GCGATCATGGAACAAGGAACT
	Shavenbaby_R	CGCCAGAGAAACGGTATTGG
NVE24711	Dachshund_F	TCCTCACCATCCACAAACTCC
	Dachshund_R	TCTGAATCTCGCCTCTTGCC
NVE11868	NVE11868_F	ATACGACAAGAGGCCACGCA
	NVE11868_R	TAAATGCTCTCCCGCACTCC
NVE14608	FoxJ1_F	CGTACGCGACGCTTATATGTATGG
	FoxJ1_R	CTGTTAGTCGAAATGAGCTGCTTCAG
NVE4967	Not-like_F	TTACCACCCACAAGCTAACGG
	Not-like_R	TTATCGAACGTTCTGCATCCC
NVE21292	Rough_F	GAAACATGCAAAGTCCCTCCTTC
	Rough_R	CGGTACAGGACTCGCCATAGAT
NVE15777	Sox14-like_F	GAAAAGACGCGCCATGAACA
	Sox14-like_R	CGTTAAGCCAACGGACCTCA
NVE17371	FoxQ2d_F	CGCGATAGATCAAGCTAAGACCG
	FoxQ2d_R	CATAGATTGTTCGCCAGTGCGT
NVE16639	GFI_F	TCCGAGTGCAAAACTGAGGG
	GFI_R	TCATTAGAAGAGCCCCGCTG
NVE1324	FoxL2_F	TGCCACTACATTGGACACCG
	FoxL2_R	TGCGGAGAAAGATACGAGACAAG
NVE4006	DMRT_F	TGCCTTGTACCATCGCATCC
	DMRT_R	ATTTCTGGCCACATAGGGCG
NVE20898	Nk5_F	TCACAGGCTTAAAACGTCGC
	Nk5_R	ACCTGCTATTTGACCTACACCA
NVE14554	DRGX_F	GCGAGTGTTTCTGCTCAACC
	DRGX_R	ACTGGATGCAAGCTCTTTTGTTT

Supplementary Table 5 (continued)		
NVE21395	Sp1-4_F	GCATGGAAAAACTCGTCCACT
	Sp1-4_R	TTCCAGTTCCTCGCCAATGC
NVE5430	PaxB_F	ACGGGCTCCATAAAACCTGG
	PaxB_R	CATTGAGCGGGAACAGCAAA
NVE7655	NVE7655_F	AGTGCGTCAGCAAGAGTGTC
	NVE7655_R	CCATCAACTGCAGCAACGTC
NVE20892	MsxA_F	ATTCACAGCAGGTTAGGCCC
	MsxA_R	ACGCTCAAAACGATGGCTCT
NVE20899	Nk1_F	ACACGGAGGGCCAGTAAGTA
	Nk1_R	ACAGCTCTTGACTGTTGGGA
NVE8363	Dlx_F	TGGCGTGTTGATAAGGCCCT
	Dlx_R	GCGTCGAGAATCAGCGATGA
NVE21445	Rx_F	ACGCTGGCCATCATGTATACT
	Rx_R	CACGTATTAGTGCCCGGGAT
NVE14243	HES-like_F	AGCCTCTCTTATGCCTACTCAG
	HES-like_R	TAAGGCTCATGTACGAAAGCA
NVE6876	FoxG1_F	TGGCTTCAAACACACAACAGT
	FoxG1_R	AACGCTGACATACCCTCTTGG
no NVE model	SP6-9_F	ATGCTAGCTGCAACTTGTAGT
	SP6-9_R	TCATGAGTCACTTAGAATGCGCG
NVE16658	APC_F	GAAAGCAGAGCGGAAACAAC
	APC_R	AATTCTCAATCGACGCCATC

Supplementary References

1 Letunic, I. & Bork, P. 20 years of the SMART protein domain annotation resource. *Nucleic Acids Res* **46**, D493-D496 (2017).

2 Kraus, Y., Aman, A., Technau, U. & Genikhovich, G. Pre-bilaterian origin of the blastoporal axial organizer. *Nat Commun* **7**, 11694 (2016).

3 Leclère, L., Bause, M., Sinigaglia, C., Steger, J. & Rentzsch, F. Development of the aboral domain in Nematostella requires beta-catenin and the opposing activities of Six3/6 and Frizzled5/8. *Development* **143**, 1766-1777 (2016).

4 Kirillova, A. *et al.* Germ-layer commitment and axis formation in sea anemone embryonic cell aggregates. *Proc Natl Acad Sci USA* **115**, 1813-1818 (2018).

5 Servetnick, M. D. *et al.* Cas9-mediated excision of *Nematostella brachyury* disrupts endoderm development, pharynx formation and oral-aboral patterning. *Development* **144**, 2951-2960 (2017).

6 Sinigaglia, C., Busengdal, H., Lecle` re, L., Technau, U., Rentzsch, F. The Bilaterian Head Patterning Gene six3/6 Controls Aboral Domain Development in a Cnidarian. *PLoS biology* **11**, e1001488 (2013).
