# Supplementary material for: Cnidarian-bilaterian comparison reveals the ancestral regulatory logic of the β-catenin dependent axial patterning
Source: Nat Commun. 2021 Jun 29;12:4032. doi: 10.1038/s41467-021-24346-8 (PMC8241978; doi:10.1038/s41467-021-24346-8)
Supplement: Supplementary file 3 — Reporting Summary [file 41467_2021_24346_MOESM3_ESM.pdf]

nature research

Corresponding author(s): Grigory Genikhovich

Last updated by author(s): May 8, 2021

Reporting Summary

Nature Research wishes to improve the reproducibility of the work that we publish. This form provides structure for consistency and transparency in reporting. For further information on Nature Research policies, see our Editorial Policies and the Editorial Policy Checklist.

Statistics

For all statistical analyses, confirm that the following items are present in the figure legend, table legend, main text, or Methods section.

n/a	Confirmed	
☐	☒	The exact sample size (*n*) for each experimental group/condition, given as a discrete number and unit of measurement
☐	☒	A statement on whether measurements were taken from distinct samples or whether the same sample was measured repeatedly
☐	☒	The statistical test(s) used AND whether they are one- or two-sided<br>*Only common tests should be described solely by name; describe more complex techniques in the Methods section.*
☒	☐	A description of all covariates tested
☐	☒	A description of any assumptions or corrections, such as tests of normality and adjustment for multiple comparisons
☐	☒	A full description of the statistical parameters including central tendency (e.g. means) or other basic estimates (e.g. regression coefficient) AND variation (e.g. standard deviation) or associated estimates of uncertainty (e.g. confidence intervals)
☐	☒	For null hypothesis testing, the test statistic (e.g. *F*, *t*, *r*) with confidence intervals, effect sizes, degrees of freedom and *P* value noted<br>*Give P values as exact values whenever suitable.*
☒	☐	For Bayesian analysis, information on the choice of priors and Markov chain Monte Carlo settings
☒	☐	For hierarchical and complex designs, identification of the appropriate level for tests and full reporting of outcomes
☒	☐	Estimates of effect sizes (e.g. Cohen's *d*, Pearson's *r*), indicating how they were calculated

Our web collection on statistics for biologists contains articles on many of the points above.

Software and code

Policy information about availability of computer code

Data collection	RNA-Seq data were obtained from Illumina HiSeq 2500 platform with SR50 mode
Data analysis	SAMtools 1.11, STAR2.5.3a, featureCounts v1.6.2, DESeq2 1.14.1, Interproscan 5.7-48.0

For manuscripts utilizing custom algorithms or software that are central to the research but not yet described in published literature, software must be made available to editors and reviewers. We strongly encourage code deposition in a community repository (e.g. GitHub). See the Nature Research guidelines for submitting code & software for further information.

Data

Policy information about availability of data

All manuscripts must include a data availability statement. This statement should provide the following information, where applicable:

- Accession codes, unique identifiers, or web links for publicly available datasets
- A list of figures that have associated raw data
- A description of any restrictions on data availability

All data needed to evaluate the conclusions in the paper are present in the paper or the supplementary materials. Raw RNA-seq reads have been deposited in the NCBI BioProject database under the accession code: PRJNA661731 (https://www.ncbi.nlm.nih.gov/bioproject/PRJNA661731).

Field-specific reporting

Life sciences study design

All studies must disclose on these points even when the disclosure is negative.

Sample size	No specific sample size calculation was performed. The number of embryos taken per biological replicate of each treatment used for subsequent RNASeq was estimated by eye to be always over 300. This number of embryos yields sufficient amount of RNA for Illumina library preparation.
Data exclusions	no data was excluded
Replication	the number of biological replicates for RNASeq treatment varied between 3 and 6, all knockdown experiments were replicated at least three times. All attempts at replication were successful. All other experiments were replicated at least twice. Only biological replicates were used.
Randomization	the embryos were randomly chosen from the single spawn and subsequently grouped by treatment
Blinding	No blinding was performed. The outcomes of the experiments were evaluated by at least two independent researchers.

Reporting for specific materials, systems and methods

We require information from authors about some types of materials, experimental systems and methods used in many studies. Here, indicate whether each material, system or method listed is relevant to your study. If you are not sure if a list item applies to your research, read the appropriate section before selecting a response.

Materials & experimental systems

n/a	Involved in the study	
☐	☒	Antibodies
☒	☐	Eukaryotic cell lines
☒	☐	Palaeontology and archaeology
☐	☒	Animals and other organisms
☒	☐	Human research participants
☒	☐	Clinical data
☒	☐	Dual use research of concern

Methods

n/a	Involved in the study	
☒	☐	ChIP-seq
☒	☐	Flow cytometry
☒	☐	MRI-based neuroimaging

Antibodies

Antibodies used	mouse monoclonal anti-acetylated tubulin (Sigma T6793), Alexa Fluor 568 rabbit anti-mouse IgG (Molecular Probes A-11061), anti-Digoxigenin-AP Fab fragments (Roche 11093274910), anti-Digoxigenin-POD Fab fragments (Roche 11633716001), anti-Fluorescein-POD Fab fragments (Roche 11426346910)
Validation	Mouse monoclonal anti-acetylated tubulin (Sigma T6793) web page: https://www.sigmaaldrich.com/catalog/product/sigma/t6793<br>Previous use for immunohistochemistry in Nematostella: Genikhovich and Technau (2009) Anti-acetylated Tubulin Antibody Staining and Phalloidin Staining<br>in the Starlet Sea Anemone Nematostella vectensis. Cold Spring Harb Protoc; 2009; doi:10.1101/pdb.prot5283; Richards and Rentzsch (2014) Transgenic analysis of a SoxB gene reveals neural progenitor cells in the cnidarian Nematostella vectensis. Development 141, 4681-4689, doi:10.1242/dev.112029.<br>Alexa Fluor 568 rabbit anti-mouse IgG (Molecular Probes A-11061) web page: https://www.thermofisher.com/antibody/product/Rabbit-anti-Mouse-IgG-H-L-Cross-Adsorbed-Secondary-Antibody-Polyclonal/A-11061 (see also for references)<br>Anti-Digoxigenin-AP Fab fragments (Roche 11093274910) web page: https://www.sigmaaldrich.com/catalog/product/roche/11093274910<br>Previous use for in situ hybridization in Nematostella: Kraus et al. (2016) Pre-bilaterian origin of the blastoporal axial organizer. Nat. Comm. 27, 11694,<br>doi: 10.1038/ncomms11694.<br>Anti-Digoxigenin-POD Fab fragments (Roche 11633716001) web page: https://www.sigmaaldrich.com/catalog/product/roche/11633716001<br>Previous use for fluorescent situ hybridization in Nematostella: Steinmetz et al. (2017) Gut-like ectodermal tissue in a sea anemone challenges germ layer homology. Nat. Ecol. Evol 1, 1535-1542, doi: 10.1038/s41559-017-0285-5.<br>Anti-Fluorescein-POD Fab fragments (Roche 11426346910) web page: https://www.sigmaaldrich.com/catalog/product/roche/11426346910<br>Previous use for fluorescent situ hybridization in Nematostella: Steinmetz et al. (2017) Gut-like ectodermal tissue in a sea anemone challenges germ layer homology. Nat. Ecol. Evol 1, 1535-1542, doi: 10.1038/s41559-017-0285-5.

Animals and other organisms

Policy information about studies involving animals; ARRIVE guidelines recommended for reporting animal research

Laboratory animals	Nematostella vectensis (Cnidaria, Anthozoa), University of Vienna laboratory strain. Male and female adults of undetermined age were spawned to produce embryos, which were used for the experiments at the age of 0 to 10 days. The sex of the embryos was not determined.
Wild animals	the study did not involve wild animals
Field-collected samples	the study did not involve field-collected samples
Ethics oversight	No ethics approval is required for work with cnidarians according to Austrian law

Note that full information on the approval of the study protocol must also be provided in the manuscript.
